# Evolutionary patterns and research frontiers in autophagy in podocytopathies: a bibliometric analysis

**DOI:** 10.3389/fmed.2024.1445550

**Published:** 2025-01-09

**Authors:** Feng Liu, Ziyu Xu, Gaijie Chen, Xiaojing Xu, Huixia Cao, Jiefang Chen

**Affiliations:** ^1^Department of Nephrology, Henan Provincial People's Hospital, People's Hospital of Zhengzhou University, Zhengzhou, China; ^2^Health Management Center, Henan Provincial People's Hospital, People's Hospital of Zhengzhou University, Zhengzhou, China; ^3^Department of Respiratory and Critical Care Medicine, Henan Provincial People's Hospital, People's Hospital of Zhengzhou University, Zhengzhou, China; ^4^Department of Neurology, Henan Provincial People's Hospital, People's Hospital of Zhengzhou University, Zhengzhou, China

**Keywords:** bibliometric analysis, podocytopathies, autophagy, visualization analysis, Web of Science Core Collection

## Abstract

**Introduction:**

Podocytopathies are a uniquely renal disease syndrome, in which direct or indirect podocyte injury leads to proteinuria or nephrotic syndrome. Of the many factors that contribute to podocytopathies, the abnormal regulation of autophagy, such insufficient or excessive autophagy levels, have been proposed to play a significant role in the occurrence and development of podocytopathies. However, there still has been a lack of systematic and comparative research to elucidate exact role of autophagy in podocytopathies and its current research status. This study aims to utilize bibliometric analysis to clarify the role of autophagy in the pathogenesis of podocytopathies, analyze the research focus in this area, as well as explore the future research trends.

**Methods:**

We retrieved original articles and review papers with respect to autophagy in podocytopathies research published between the year 2008 and 2022 from the Web of Science Core Collection (WOSCC). Then, VOSviewer and CiteSpace software were employed to reveal the leading subjects and generate visual maps of countries/regions, organizations, authors, journals, and keyword networks in this field.

**Results and discussion:**

A total of 825 publications regarding autophagy in podocytopathies published between 2008 and 2022 were included, with China contributing the most followed by the United States and Japan. Professor Koya Daisuke, Professor He Qiang, and Professor Jin Juan are the most prolific researchers in this field. Oxidative stress, the NLRP3 inflammasome, and therapeutic targets were the knowledge base for the research in this special field. Taken together, this bibliometric analysis helps us reveal the current research hotspots and guide future research directions, which provides a reference for scholars to further investigate the role of autophagy in podocytopathies as well as conduct clinical trial with autophagy regulators in podocytopathies.

## 1 Introduction

Podocytes are highly differentiated visceral epithelial cells within the glomerulus, together with the glomerular endothelium and glomerular basement membrane (GBM), providing an important component of the glomerular filtration barrier and maintaining the integrity of the glomerular structure (1, 2). Current researches have confirmed that podocyte injury or loss contributes to massive proteinuria, glomerulosclerosis, and subsequent renal function decline (3, 4). Different genetic and environmental risk factors-induced podocyte damage represent different patterns of podocyte injury rather than defining a unique disease diagnosis that would imply a specific therapy (5–7). Thus, it is particularly important to rename this type of diseases, mainly including minimal change disease (MCD), focal segmental glomerulosclerosis (FSGS), diabetic kidney disease (DKD), lupus nephritis (LN), IgA nephropathy (IgAN), membranous nephropathy (MN), and hypertensive nephropathy (HTN), as “podocytopathies,” which prompts a diagnostic workup to identify the causative trigger or triggers of podocyte injury and so as to determine prognosis and determination of personalized treatment (3, 8). Despite reliable epidemiological data for podocytopathies is still lacking, the prevalence of podocytopathies seems to be increasing worldwide, which is the most significant cause of end-stage renal disease (ESRD) (3, 6). Over the past decades, we have witnessed dramatic advances in the understanding of podocyte biology as well as molecular mechanisms involved in podocytopathies (9, 10). However, there are currently no effective and curative treatment strategies for podocytopathies, and the standard treatment of podocytopathies mainly relies on repurposed immunosuppressive drugs (11). Meanwhile, the clinical trials of podocyte specific drug formulations and the development of podocyte-directed drug delivery systems have not yet made substantial progress (4). Therefore, the identification of novel potential therapeutic targets that could beneficially mitigate podocyte injury or recover podocyte function has great clinical importance.

Autophagy is a highly conserved process of lysosomal-dependent degradation of damaged organelles and toxic protein aggregates, which is involved in a variety of cellular biological activities (12–14). In adult kidneys, basal autophagy in resident kidney cells, including podocytes, proximal tubule epithelial cells, glomerular mesangial cells, and glomerular endothelial cells, seems to be crucial for maintaining kidney integrity and normal physiology (15, 16). Podocytes display a high level of basal autophagy, which is important for the maintenance of podocyte homeostasis (17–19). The impact of autophagy in podocytopathies is emerging as a recent research hotspot, offering novel insights into podocytopathies prevention and treatment. Accumulating evidence has indicated that autophagy plays a critical role in podocytopathies (20). For example, a study on *in vivo* animals confirmed that podocyte-specific deletion of autophagy-related 5 (Atg5) exhibited strongly increased susceptibility to models of glomerular disease, highlighting the importance of induced autophagy as a key homeostatic mechanism to maintain podocyte integrity (21). Another study has demonstrated that administration of autophagy inhibitors (3 methyladenine or chloroquine) in a puromycin aminonucleoside (PAN)-induced podocyte injury model resulted in aggravated podocytes foot process effacement and proteinuria, whereas stimulation of autophagy by rapamycin led to alleviated podocyte damage as well as decreased proteinuria (22). The above researches suggest that autophagy regulators do have protective effects on podocytopathies. However, so far, no clinical trials with autophagy regulators in podocytopathies have been successfully conducted (20). Nowadays, investigating the role of autophagy in podocytopathies still remains an area of active research. Therefore, a better understanding of autophagy in podocytopathies is of great importance for identifying novel effective approaches to prevent and treat this disease. Over the past 10 years or so, we have witnessed a growing number of researches focused on autophagy in podocytopathies (20, 23). However, the explosive growth of publications on this topic may keep researchers from fully understanding of key developments and potential future directions in the field (24). Therefore, a systemic analysis of the hot spots and trends within autophagy in podocytopathies field is very necessary. However, research in this area is currently lacking.

Bibliometrics is an emerging interdisciplinary subject that applies mathematical and statistical methods to quantitatively analyze academic literature in a designated research field (25, 26). With this method, researchers can quickly dig deeper into the evolution of subjects, major research areas, as well as future research directions in a particular research area (25, 27). As a complementary research method, bibliometrics has been widely used in many disciplines to assess global trends and hotspots in certain research area (25, 27). Whereas, there is still lacking in-depth bibliometric analysis of autophagy in podocytopathies area. Therefore, to better foster the development of autophagy in podocytopathies research, a scientifically oriented analysis using bibliometric methods is warranted to assess the current status of research and identify more valuable research directions, thus promoting the further development of this field.

In the present study, we systematically analyzed the relevant literature in the field of autophagy in podocytopathies using a bibliometric approach and conducted a systematic evaluation of the status of recent researches, current research priorities, and novel research trends, highlighting landmark results and pointing out future research directions. We anticipate that this paper will inspire researchers with novel ideas for autophagy in podocytopathies.

## 2 Methods

### 2.1 Data sources and search strategy

In this study, all the data were acquired from the Web of Science Core Collection (WoSCC) database, which is one of the most authoritative and influential databases of scientific literature in bibliometric analyses (28). The primary search terms included “podocytopathies,” “minimal change disease,” “focal segmental glomerulosclerosis,” “diabetic kidney disease,” “diabetic nephropathy,” “lupus nephritis,” “IgA nephropathy,” “membranous nephropathy,” “hypertensive nephropathy,” “autophagy,” “macroautophagy,” “microautophagy,” “chaperone-mediated autophagy,” “autophagocytosis,” “reticulophagy,” “ER-Phagy,” “nucleophagy,” “ribophagy,” “lipophagy,” and “mitophagy.” To identify studies related to autophagy in podocytopathies during the year from 2008 to 2022, we used the following search strategies: #1 (TS = (“podocytopathies” OR “minimal change disease” OR “focal segmental glomerulosclerosis” OR “diabetic kidney disease” OR “diabetic nephropathy” OR “lupus nephritis” OR “IgA nephropathy” OR “membranous nephropathy” OR “hypertensive nephropathy”)); #2 (TS = (“autophagy” OR “macroautophagy” OR “microautophagy” OR “chaperone-mediated autophagy” OR “autophagocytosis” OR “reticulophagy” OR “ER-Phagy” OR “nucleophagy” OR “ribophagy” OR “lipophagy” OR “mitophagy”)); #3 (#1 and #2). To avoid any bias caused by the ongoing update of WOS database (29), the necessary publications were retrieved only once by three independent researchers on 20 March 2023. Only “research articles” and “review articles” published in English were considered for inclusion. Overall, 825 literature sources were analyzed, including 614 original research articles and 211 review articles.

### 2.2 Data analysis and visualization

VOSviewer and CiteSpace software are indispensable tools for researchers and academicians in the investigation of scholarly data, which can complement each other and provide a comprehensive toolkit for scholars to obtain a deeper understanding of academic literature, collaboration patterns, and current progress in a certain research field (30). First and foremost, the data we collected in the field of autophagy in podocytopathies met the minimum sample size requirements for bibliometric analysis (more than 200 articles) (31). Then, bibliometric analysis and data visualization were systematically conducted with VOSviewer software (version 1.6.19) and CiteSpace software (version 6.2.R2 Basic) as described in our previously study (32). Specifically, VOSviewer software was used to generate knowledge maps of the citation networks among countries/regions, organizations, authors, co-citation authors, co-occurrence networks, etc. Additionally, CiteSpace software was employed to obtain visualization knowledge maps of cooperation among institutes, dual-map overlays of journals, cluster views of co-cited references, top references with the strongest citation bursts, and top keywords with strong citation bursts in the field of autophagy in podocytopathies. Overall, those two software programs were employed for network visualization and analysis to gain insights into the field of autophagy in podocytopathies, as well as discover the research frontier and subsequent trends in this special field using a considerable quantity of data.

## 3 Results

### 3.1 Annual publication outputs

**Figure 1:**
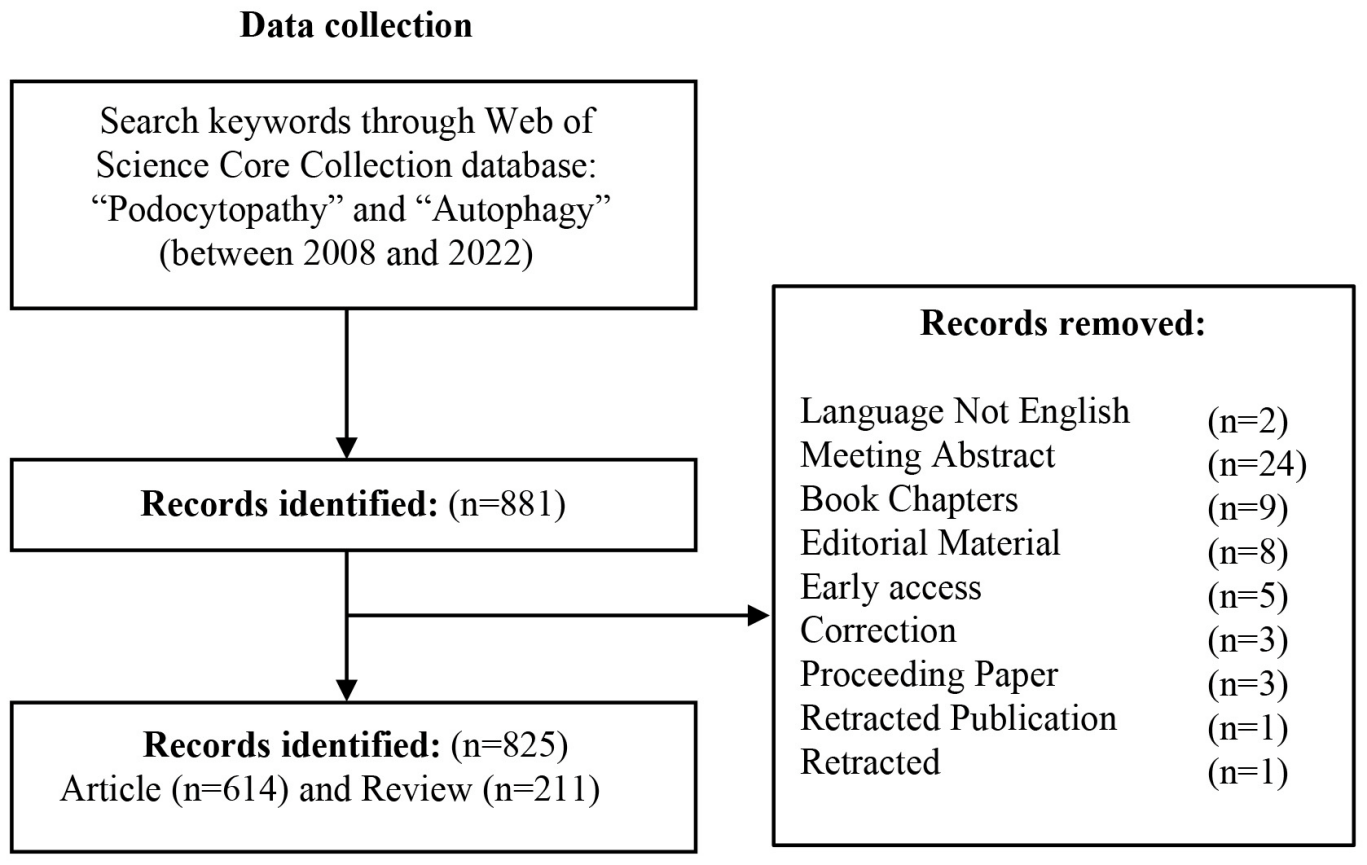
Flow chart displaying the literature selection with respect to autophagy in podocytopathy researches.

**Figure 2:**
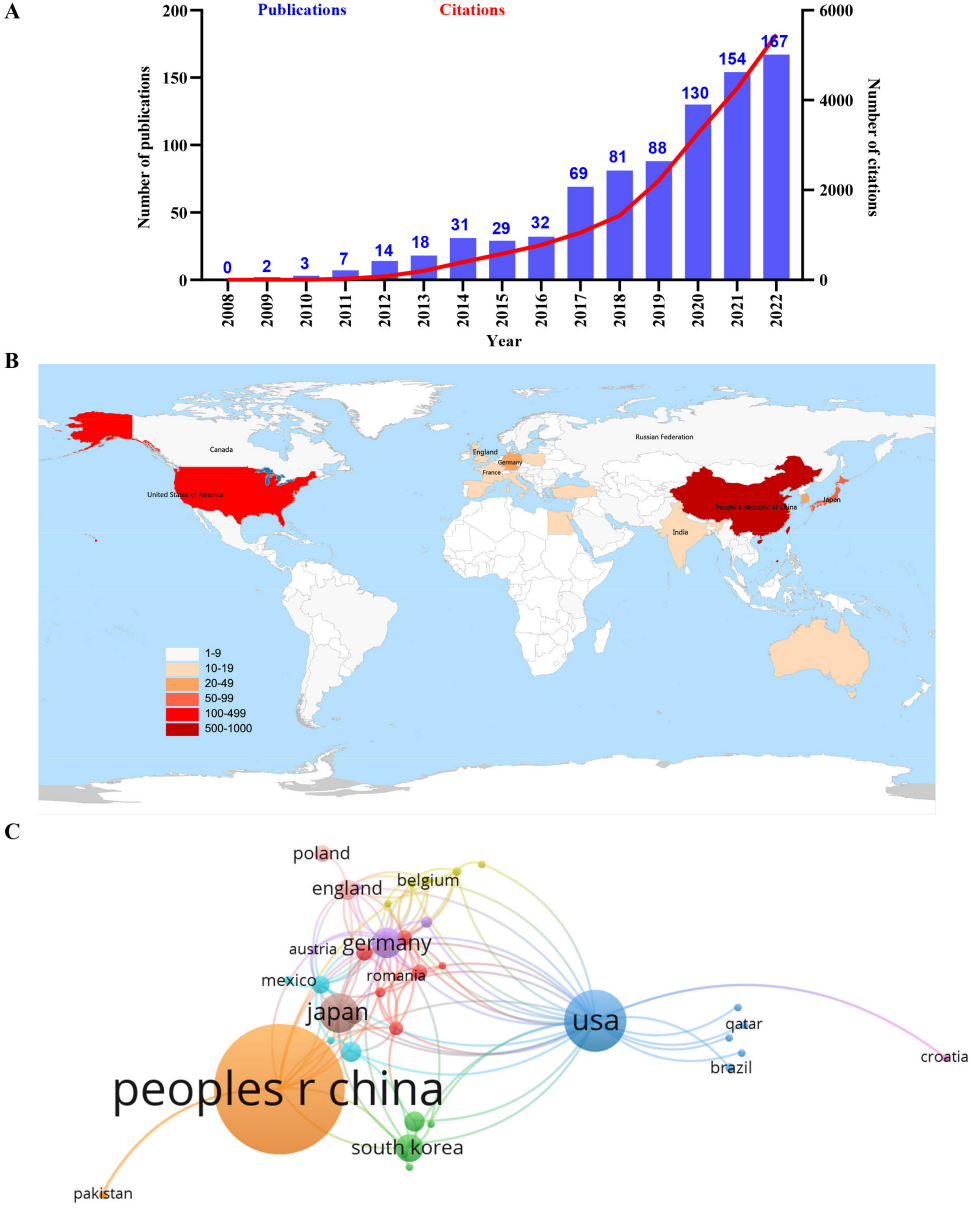
Leading countries in the field of autophagy in podocytopathy. **(A)** The annual number and total citations of articles related to autophagy in podocytopathy from 2008 to 2022. **(B)** Geographical distribution of global output. **(C)** Visual cluster analysis of cooperation among countries. The links between different countries represented the frequency of the collaborations in the field of autophagy in podocytopathy.

As illustrated in Figure 1, according to the flow chart of the retrieval process, a total of 825 publications on autophagy in podocytopathies area published during 2008 and 2022 were included, of which 614 original research articles accounted for 74.42% of the total publications and 211 review articles accounted for 25.58%. The dynamic change in the total number of publications during the past 15 years can reflect the overall development trend in this special field. The number of publications per year increased steadily from 2008 to 2022, increasing from < 5 papers before the year 2010 to more than 100 papers after the year 2019 (Figure 2A), indicating that autophagy has now become a new focus of research in the field of podocytopathies. Although the annual growth rate of publications fluctuated over the past decade, it still maintained an average annual growth rate of 25.07% (Figure 2A). Correspondingly, the number of citations per year steadily boosted from < 100 before 2013 to more than 1,000 for the first time in 2017, and then surged to 5,456 in 2022 (Figure 2A).

### 3.2 Contribution of countries/regions

During the past 15 years, 48 countries/regions have successively conducted research on the field of autophagy in podocytopathies, among which China ranked first globally in output with 540 publications, followed by the United States and Japan with 128 and 56 papers, respectively (Figure 2B, Table 1). Over 70% of the global publications were from China, the United States, and Japan, indicating that these three countries contributed the most to research on autophagy in podocytopathies field. In addition, Germany, Korea, the United Kingdom, Egypt, and India published more than 100 publications as well, also making important contributions to this field (Table 1). It is worth noting that the ranking of average citations per item (ACI) is markedly different from the ranking based on publication quantity, with France having the highest ACI (ACI = 56.08), followed by Australia (ACI = 50.58), and Germany (ACI = 48.88) (Table 1). However, the ACI of the top three countries in terms of publication volume are 18.75 (China), 44.21 (the United States), and 46.45 (Japan), respectively (Table 1). In the VOSviewer network maps, different color nodes indicate different countries/regions and a larger node represents more publications in the country/region. The links represent the collaborative relationships between different countries/regions involved in research on autophagy in podocytopathies. Specifically, among the top 5 countries/regions in terms of publications, the United States and Germany cooperated most closely with other countries/regions, while China, Japan, and Korea had little cooperation with others (Figure 2C). The differences in the degree of cooperation among different countries/regions may be related to different academic atmospheres in Europe, America, and Asia, which still needs further investigation.

**Table 1:** Top 10 productive countries regarding the research on autophagy in podocytopathy.

**Rank**	**Country**	**Publications**	**Percentage (%)**	**ACI**	**H-index**
1	China	540	54.32%	18.75	47
2	United States	128	12.88%	44.21	42
3	Japan	56	5.63%	46.45	26
4	Germany	34	3.42%	48.88	19
5	Korea	30	3.02%	28.77	15
6	United Kingdom	18	1.81%	27.95	10
7	Egypt	16	1.61%	18.81	9
8	India	16	1.61%	18.19	8
9	France	13	1.31%	56.08	8
10	Australia	12	1.21%	50.58	8

ACI, Average citation per item.

### 3.3 Contribution of organizations

**Figure 3:**
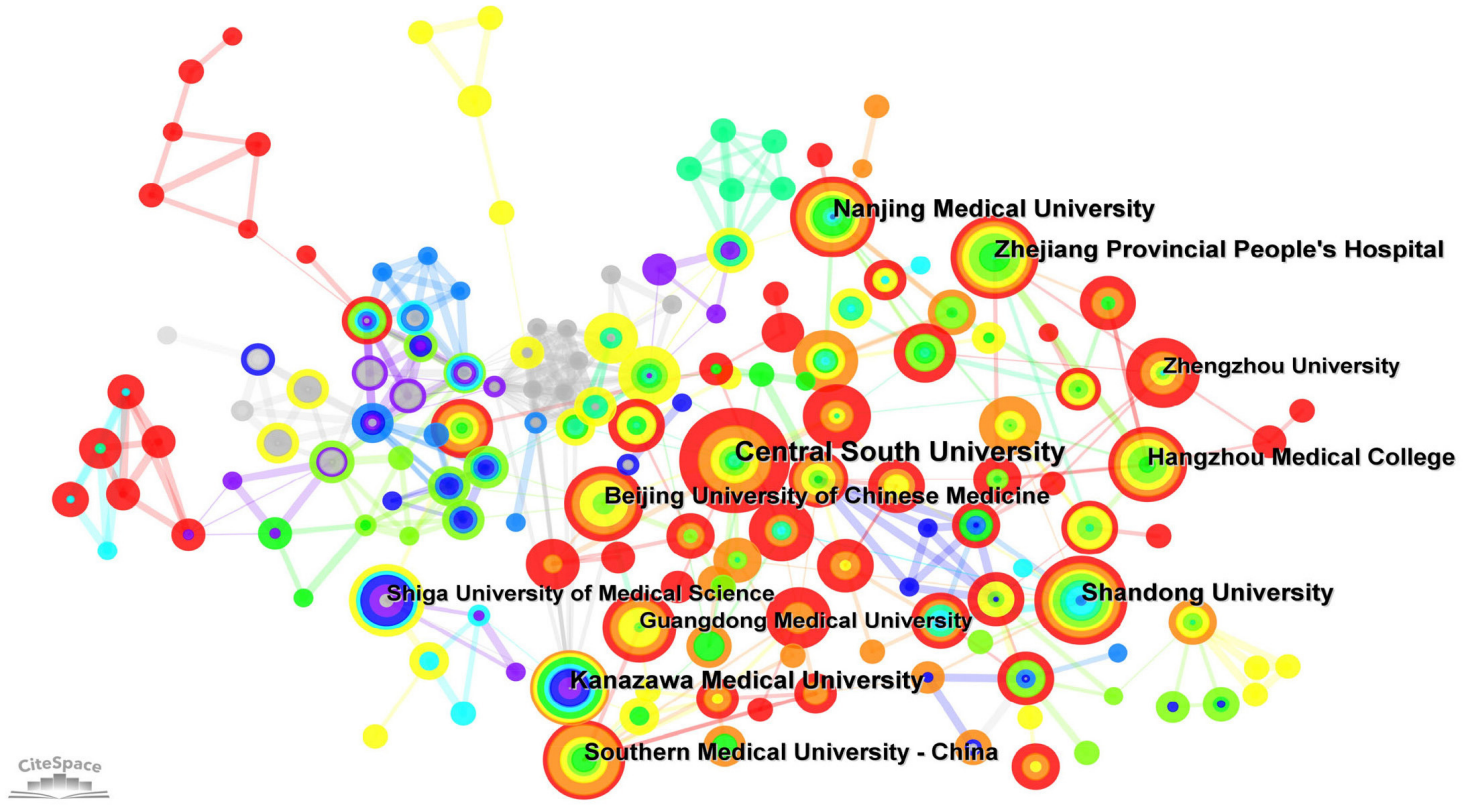
Visualization of active institutes. Cluster analysis of cooperation among institutes generated by CiteSpace in the field of autophagy in podocytopathy.

Over the past 15 years, 928 organizations have contributed to the development of the field of autophagy in podocytopathies, and the collaborative relationships between them are visualized in Figure 3. Among these organizations, Shandong University ranked first with 28 publications, followed by Central South University and Nanjing Medical University with 27 and 25 publications, respectively (Table 2). In addition, Kanazawa Medical University (24 publications), Zhejiang Provincial People's Hospital (22 publications), Beijing University of Chinese Medicine (21 publications), Southern Medical University (20 publications), and Shiga University of Medical Science (19 publications) also contributed greatly to the research in this field (Table 2). It is worth noting that among the top ten organizations, 8 belonged to China and 2 belonged to Japan, indicating that these two countries have dominated research on “autophagy in podocytopathies” over the past 15 years. In terms of cooperation between institutions, Shanghai Jiao Tong University (0.12), the University of Freiburg (0.08), Central South University (0.08), and the Chinese Academy of Sciences (0.08) were the core nodes with relatively higher centrality values. Yet it's worth noting that even if the three institutions mentioned above are at the core of institutional cooperation, the centrality values are still in very low levels. Therefore, global cooperation should be strengthened in “autophagy in podocytopathies” research, so as to contribute to the development of this field.

**Table 2:** Top 10 productive organizations regarding the research on autophagy in podocytopathy.

**Rank**	**Organization**	**Publications**	**Citations**	**Countries**
1	Shandong Univ	28	764	China
2	Cent South Univ	27	517	China
3	Nanjing Med Univ	25	853	China
4	Kanazawa Med Univ	24	1,745	Japan
5	Zhejiang Prov Peoples Hosp	22	497	China
6	Beijing Univ Chinese Med	21	294	China
7	Southern Med Univ	20	406	China
8	Shiga Univ Med Sci	19	1,473	Japan
9	Zhengzhou Univ	18	252	China
10	Guangdong Med Univ	17	321	China

### 3.4 Contributions of authors and co-cited authors

**Figure 4:**
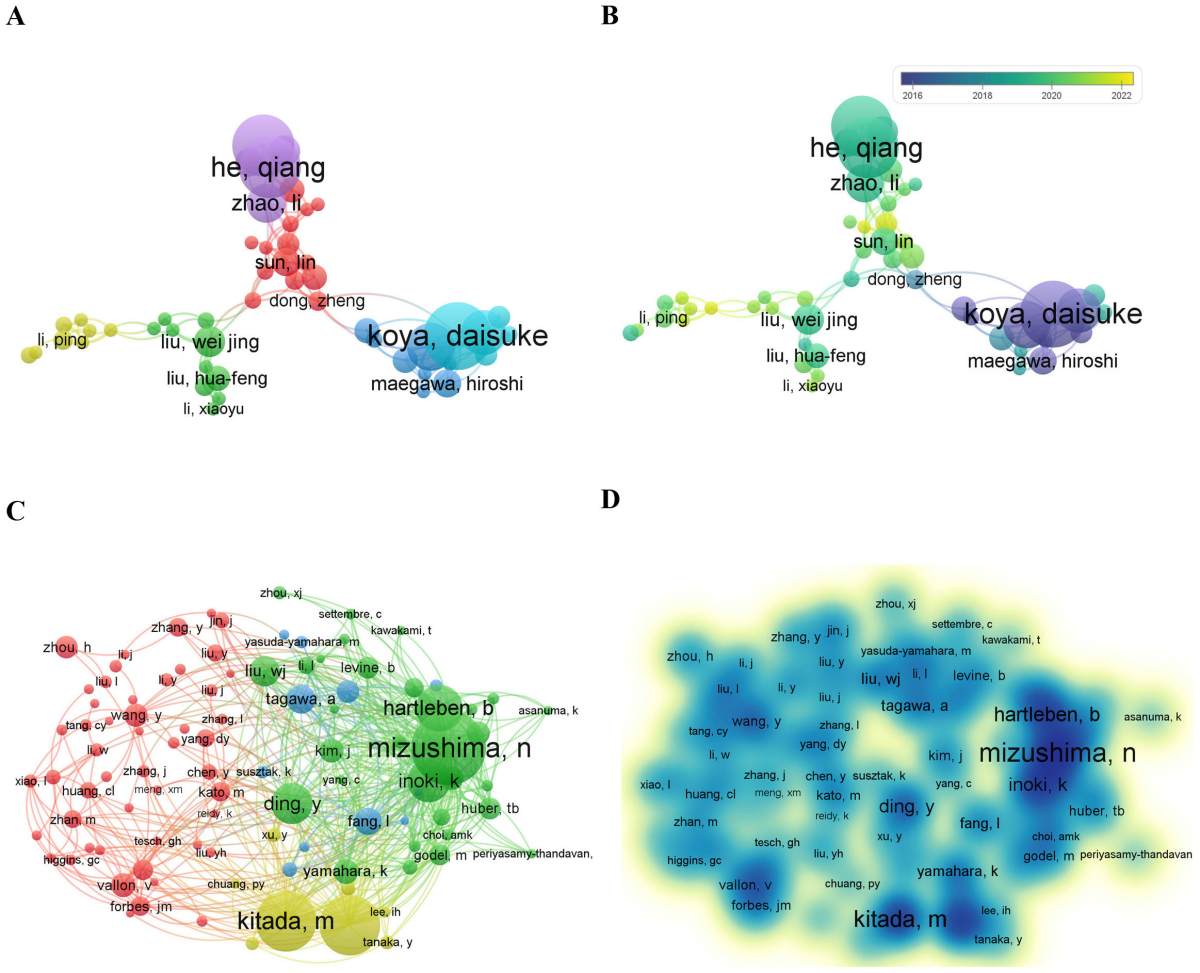
The collaboration of authors and co-cited authors in the field of autophagy in podocytopathy. **(A)** Cooperation network of authors. Among the total 4,338 authors, 50 authors had published at least 6 papers. **(B)** Overlay visualization by time (year). Purple represented 2016 and before, green represented the year around 2018, yellow represented the year of 2022. **(C)** Co-citation network of authors. Of the 22,647 co-cited authors, 100 had at least 35 citations. **(D)** VOSviewer density visualization of the co-cited authors.

A total of 4,338 authors participated in related research on autophagy in podocytopathies in the last 15 years. Table 3 shows the detailed information of the top 10 authors who were identified as the most productive authors. The aforementioned results showed that 6 of them were from China, and the other 4 were from Japan. Similarly, the authors with the highest number of publications were Koya Daisuke (24 papers) from Kanazawa Medical University, followed by He Qiang and Jin Juan, both from Zhejiang Provincial People's Hospital with 22 publications (Table 3). According to the closeness of connections among the top 50 most productive authors, their cooperations were divided into 5 main clusters (Figure 4A). The colors in the overlay visualization shown in Figure 4B indicate the average years in which the authors published their papers. As can be seen from the overlay visualization map, the top 3 most productive authors had published their articles before 2020, indicating that their focus may have shifted away from this field in recent 3 years.

**Table 3:** Top 10 productive authors regarding the research on autophagy in podocytopathy.

**Rank**	**Authors**	**Documents**	**Citations**	**Organizations**
1	Koya Daisuke	24	1,745	Kanazawa Med Univ
2	He Qiang	22	465	Zhejiang Prov People's Hosp
3	Jin Juan	22	465	Zhejiang Prov People's Hosp
4	Kume Shinji	17	1,413	Shiga Univ Med Sci
5	Kitada Munehiro	16	841	Kanazawa Med Univ
6	Li Yiwen	11	291	Zhejiang Prov People's Hosp
7	Liu Wei Jing	11	268	Beijing Univ Chinese Med
8	Gong Jianguang	10	235	Zhejiang Prov People's Hosp
9	Maegawa Hiroshi	10	781	Shiga Univ Med Sci
10	Sun Lin	10	555	Cent South Univ

The top 10 co-cited authors are listed in Table 4. Noboru Mizushima from Tokyo Medical and Dental University was the most citated author with 301 citations, followed by Kitada Munehiro from Kanazawa Medical University and Kume Shinji from Shiga University of Medical Science, both with 244 citations. Notably, the top three co-cited authors were all from Japan, indicating that Japan played a critical role in the field of autophagy in podocytopathies. In addition, co-cited authors from Germany, the United States, and China also made important contributions to this field. The co-citation relationship network of authors with no < 35 citations obtained by the VOSviewer software showed three clusters, displaying the authors with a significant influence in these fields (Figure 4C). Furthermore, the influence of co-citation authors in the related fields is also displayed in the density map (Figure 4D).

**Table 4:** Top 10 co-citated authors in the field of autophagy in podocytopathy.

**Rank**	**Co-citated authors**	**Citations**	**Organizations**	**Countries**
1	Noboru Mizushima	301	Tokyo Med and Dent Univ	Japan
2	Kitada Munehiro	244	Kanazawa Med Univ	Japan
3	Kume Shinji	244	Shiga Univ Med Sci	Japan
4	Hartleben Bjoern	190	Hannover Med Sch	Germany
5	Inoki Ken	173	Univ Michigan	USA
6	Ding Yan	168	Weill Cornell Med Coll	USA
7	Wenjing Liu	122	Beijing Univ Chinese Med	China
8	Atsuko Tagawa	116	Shiga Univ Med Sci	Japan
9	Fang Li	110	Nanjing Med Univ	China
10	Yamahara Kosuke	102	Shiga Univ Med Sci	Japan

### 3.5 Journals and co-cited journals

A total of 285 journals reported studies on autophagy in podocytopathies, and the top ten journals with impact factors (IFs) and journal citation reports (JCRs) are shown in Table 5. The Journal of Frontiers in Pharmacology and Cell Death & Disease were the two most productive journals, while the American Journal of Physiology-Renal Physiology held the first in terms of total citations and citations per publication. Among the top ten journals, 6 journals have high impact journals (journals with an IF ≥ 5 in 2022), including Frontiers in Pharmacology (5.99, Q1), Cell Death & Disease (9.71, Q1), International Journal of Molecular Sciences (6.21, Q2), Scientific Reports (5.00, Q2), Life Sciences (6.78, Q1), and Biomedicine & Pharmacotherapy (7.42, Q1).

**Table 5:** Top 10 productive journals in the field of autophagy in podocytopathy.

**Rank**	**Journal**	**Documents**	**Citations**	**IF**	**JCR**
1	Front Pharmacol	33	348	5.99	Q1
2	Cell Death Dis	17	532	9.71	Q1
3	Am J Physiol-Renal	16	882	4.1	Q2
4	Int J Mol Sci	16	289	6.21	Q2
5	Sci Rep	16	545	5	Q2
6	Biochem Bioph Res Co	15	270	3.32	Q3
7	Life Sci	15	172	6.78	Q1
8	Biomed Pharmacother	14	177	7.42	Q1
9	Exp Ther Med	14	118	2.75	Q4
10	Mol Med Rep	14	131	3.42	Q3

**Figure 5:**
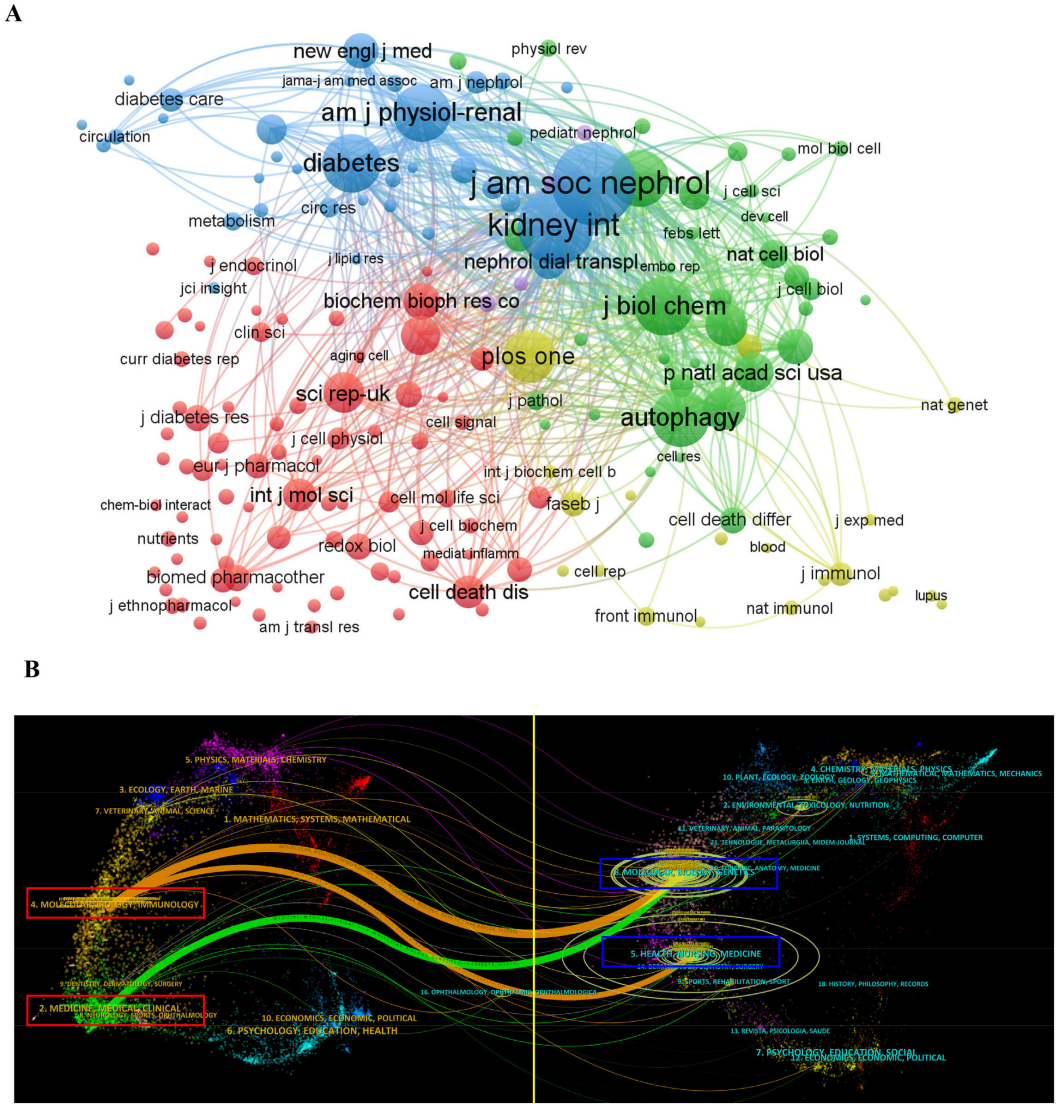
Journal analyses in the field of autophagy in podocytopathy. **(A)** VOSviewer network visualization map for co-citation analysis of active journals. Nodes size represents co-citation frequency, and the links between nodes indicate co-citation relationships. **(B)** The dual-map overlay of journals related to autophagy in podocytopathy. The labels indicate different research subjects covered by the citing journals and cited journals. Left side of the map includes the citing journals and the other side includes the cited journals.

As the influence of a journal in a research field mainly depends on its numbers of citation, the citation analysis of a journal could reflect the actual distribution of the knowledge base and important research findings in a specific field (33). Thus, co-citation analysis of VOSviewer was then carried out to analyze the co-cited journals (Figure 5A). Among the co-cited journals, there were 33,354 citations overall, and the leading 10 journals with the most influential publications in this field were cited 12,639 times, accounting for 37.89% of the total citations. The Journal of the American Society of Nephrology ranked first with 2,304 citations, followed by Kidney International (1,920 times), the Journal of Biological Chemistry (1,227 times), Diabetes (1,217 times), Journal of Clinical Investigation, and American Journal of Physiology-Renal Physiology (1,184 times), indicating that these journals have high authority in the area of autophagy in podocytopathies research. Figure 5B illustrates a dual-map overlay of the journals in the field of autophagy in podocytopathy-related imaging research. It could be seen that there were three main citation paths in the dual-map. Specifically, the published studies mainly targeted journals in the fields of “Molecular, Biology, Immunology” and “Medicine, Medical, Clinical,” whereas the most cited publications originated from the journals of “Molecular, Biology, Genetics” and “Health, Nursing, Medicine” fields.

### 3.6 Analysis of co-cited references

**Figure 6:**
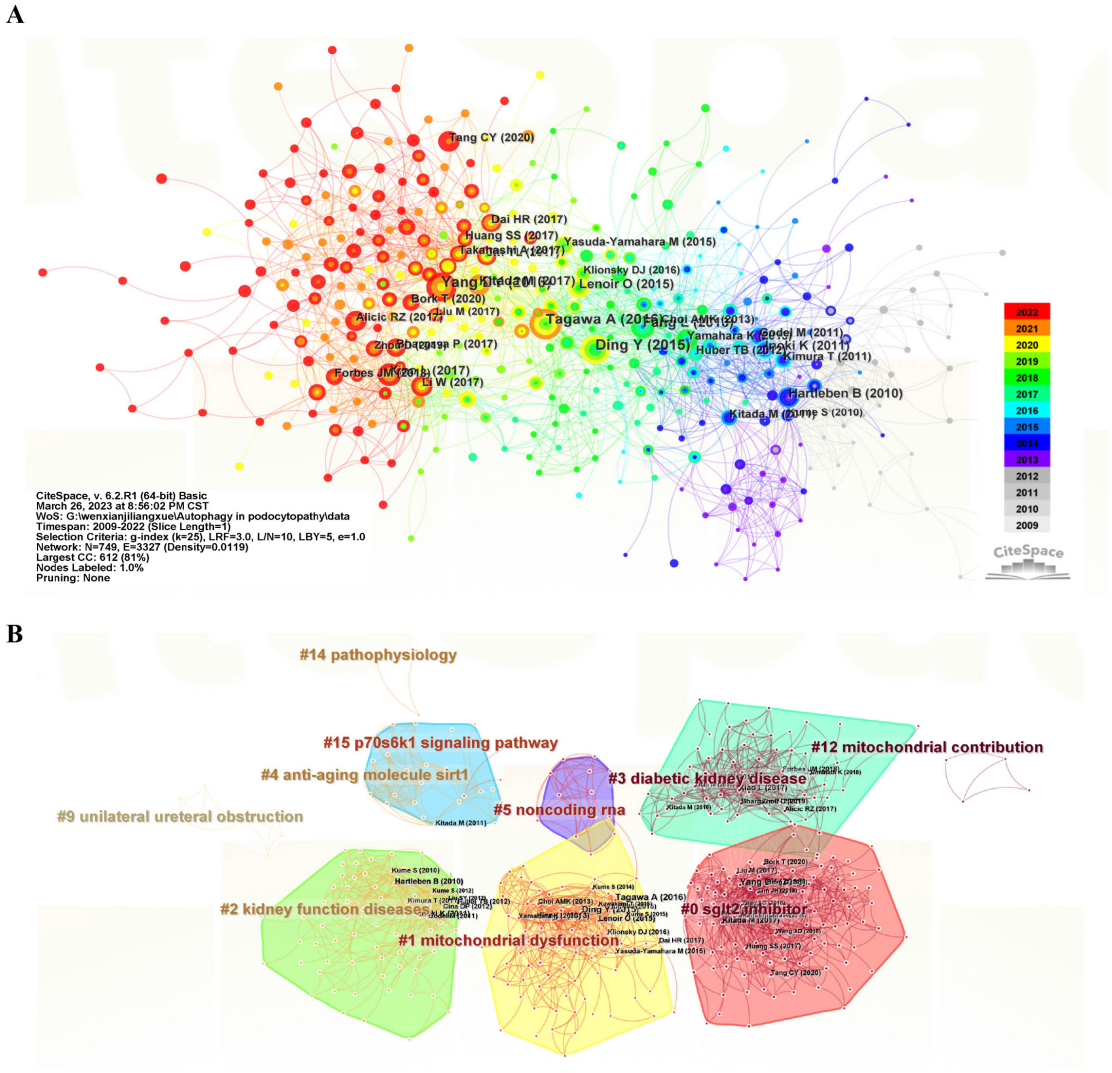
Visualization of co-cited reference analysis on autophagy research in podocytopathy. **(A)** The major references of the co-citation network (with the colors representing the year of publication from 2009 to 2022, with light gray being the earliest and red being the most recent). **(B)** Cluster view of co-cited references using keywords as label source.

A total of 945 co-cited references were analyzed by co-clustering, and the top 10 highly cited references are listed in Table 6 (21, 34–42). The article “Autophagy influences glomerular disease susceptibility and maintains podocyte” published in the Journal of Clinical Investigation in 2010 ranked first with co-citation counts of 175 (21). In regard to the topics across the above highly co-cited references, investigating the pathological role and therapeutic potential as well as preventing podocytopathies, especially regarding diabetic kidney disease, are the most popular, and genetic research on podocyte homeostasis is also pretty attractive. Three of the top ten co-cited publications were published in the Journal of Clinical Investigation, which is a highly respected and influential peer-reviewed medical journal. Moreover, we further conducted co-cited network and cluster analyses through employing CiteSpace software. The major references of the co-citation network are illustrated in Figure 6A, with the colors representing the year of publication from 2009 to 2022. The literature with the highest burst strength was the 2010 Hartleben B's early research on “Autophagy influences glomerular disease susceptibility and maintains podocyte homeostasis in aging mice” (21), which was the first *in vivo* exploration of the function of autophagy in podocytopathies, proposing that induced autophagy is a major protective mechanism against podocyte injury. The co-cited literature is synthesized into 16 clusters (numbered from 0 to 15 by size), among which the 9 main clusters with active co-citation relationships are as follows (Figure 6B), SGLT2 inhibitor (#0), mitochondrial dysfunction (#1), diabetic function disease (#2), diabetic kidney disease (#3), anti-aging molecule sirt1 (#4), non-coding RNA (#5), mitochondrial dysfunction (#12), pathophysiology (#14), and the p70s6k1 signaling pathway (#15).

**Table 6:** Top 10 highly cited publications in the field of autophagy in podocytopathy.

**Rank**	**Co-cited reference title**	**Journal**	**Citation**	**Year**	**IF**
1	Autophagy influences glomerular disease susceptibility and maintains podocyte homeostasis in aging mice	J Clin Invest	175	2010	19.49
2	Impaired Podocyte Autophagy Exacerbates Proteinuria in Diabetic Nephropathy	Diabetes	114	2016	9.34
3	Autophagy in diabetic nephropathy	J Endocr	110	2015	4.67
4	mTORC1 activation in podocytes is a critical step in the development of diabetic nephropathy in mice	J Clin Invest	95	2011	19.49
5	Role of mTOR in podocyte function and diabetic nephropathy in humans and mice	J Clin Invest	86	2011	19.49
6	Autophagy attenuates diabetic glomerular damage through protection of hyperglycemia-induced podocyte injury	PLoS One	85	2013	3.75
7	Autophagy in diabetic kidney disease: regulation, pathological role and therapeutic potential	Cell Mol Life Sci	71	2018	9.24
8	Endothelial cell and podocyte autophagy synergistically protect from diabetes-induced glomerulosclerosis	Autophagy	70	2015	13.39
9	Autophagy fights disease through cellular self-digestion	Nature	70	2008	69.5
10	Obesity-mediated autophagy insufficiency exacerbates proteinuria-induced tubulointerstitial lesions	J Am Soc Nephrol	69	2013	14.98

### 3.7 Analysis of keywords

**Figure 7:**
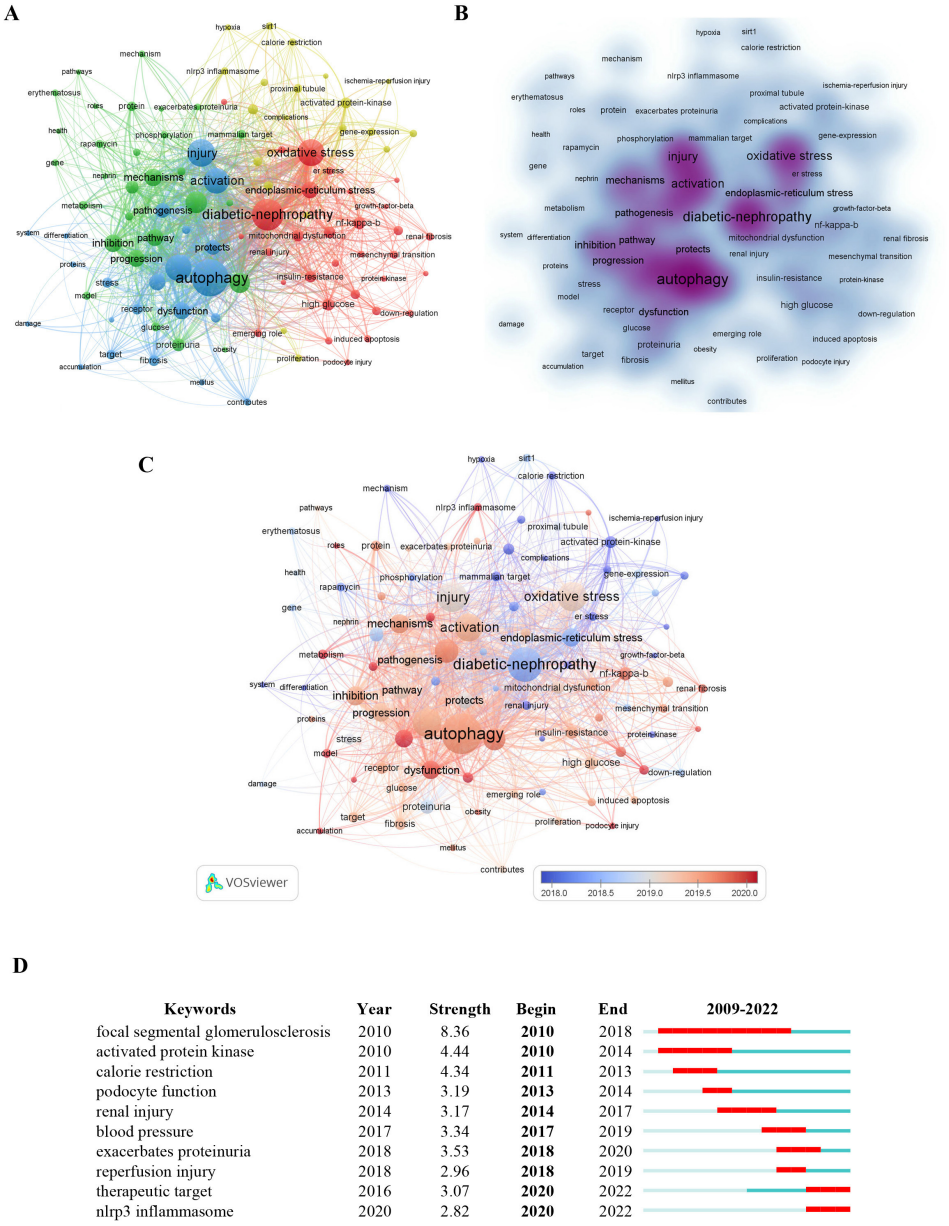
Analysis of keywords in studies related to autophagy in podocytopathy. **(A)** VOSviewer cluster visualization the 102 keywords appearing at least 10 times. **(B)** VOSviewer density visualization of the above keywords. **(C)** Keywords visualization based on the average publication year. **(D)** Top 10 keywords with the Strongest Citation Bursts.

Keyword analysis provides an overview of the current status in the field of autophagy in podocytopathies, which cloud reflect hotspots and future directions. Then, we constructed the co-occurrence network of 102 high-frequency keywords (of 1,818 in total) with at least 10 appearances per keyword and visualized it by clustering analysis. The keywords “diabetic nephropathy” (415 times), “autophagy” (247 times), “oxidative stress” (203 times), “injury” (148 times), and “endoplasmic-reticulum stress” (137 times) ranked in the top 5, suggesting that the association between autophagy and diabetic nephropathy has been one of the most prominent topics of autophagy in podocytopathies. According to Figure 7A, the green cluster is mainly consisted of “autophagy,” “mitophagy,” “damage,” “dysfunction,” “apoptosis,” etc., which are closely related to relevant mechanisms involved in programmed cell death, a finding consistent with previous report (43). The red cluster predominantly represents “oxidative stress,” “endoplasmic-reticulum stress,” “mitochondrial dysfunction,” “podocyte injury,” “chronic kidney disease,” and “diabetic nephropathy.” The above clusters mainly reflect the molecular mechanisms of podocytopathies induced by autophagy. Figure 7B is a density visualization map showing the same identification keywords, drawn according to frequency of occurrence.

Moreover, in the overlay visualization displayed in Figure 7C, keywords are colored differently according to their average publication year. Earlier keywords are displayed in blue, while red nodes represent the most recent keywords. The most recent keyword was “nlrp3 inflammasome,” followed by “mitophagy,” “podocyte autophagy,” and “inflammation,” all of which play important roles in the occurrence and development of podocytopathies. Furthermore, burst keyword detection was performed to identify the emerging concepts cited frequently in the field of autophagy in podocytopathies from 2008 to 2022, which can not only indicate the evolution of research hotpots with time but also predict the future research trend. The top 10 discovered bust keywords are shown in Figure 7D. As shown in Figure 7D, keywords such as focal segmental glomerulosclerosis (8.36, 2010–2018), podocyte function (3.19, 2013–2014), renal injury (3.17, 2014–2017), exacerbates proteinuria (3.53, 2018–2020), and reperfusion injury (2.96, 2018–2019), revealed that clarifying the etiology and pathogenesis of podocytopathies was the hotspot of autophagy research in podocytopathies over the past decade, while keywords such as therapeutic target (3.07, 2020–2022) and the NLRP3 inflammasome (2.82, 2020–2022) have become the focus of current research in this field.

## 4 Discussion

In the present study, we conducted a systematic and comprehensive bibliometric analysis of studies in the field of autophagy in podocytopathies over the past decade or so. To our knowledge, this is the first in-depth bibliometric study carried out with the aim to determine the scientific output and emerging topics related to autophagy in podocytopathies area. Our research has made a certain contribution to the field of autophagy in podocytopathies, which could guide future researchers to identify the status of recent research, the current research focus, as well as new research trends in this emerging field. Changes in yearly output are commonly considered as a crucial indicator of the development process in a certain research field (25, 44). According to the data acquired from the WoSCC database, a total of 614 original research articles and 211 reviews related to autophagy in podocytopathies were published from 2008 to 2022. Specifically, the findings of this study indicate that the annual publication output in the field of autophagy in podocytopathies displayed a continuous upward trend over the past decade or so, except for a brief setback in 2015, after which it experienced a sudden increase beginning in 2016. In general, these results reveal an increasing trend of research, indicating that an increasing number of researchers have begun to pay attention to investigating the exact role of autophagy in podocytopathies. Thus, it can be reasonably predicted that autophagy in podocytopathies will be a novel research hotspot in the foreseeable future.

When it comes to the number of publications and total citation frequency, China was the world leader in the field of autophagy in podocytopathies research, followed by the United States and Japan. According to our institutional analysis, China occupied 8 of the top 10 most productive institutions and Japan accounted for the remaining 2 according to the number of publications. At present, China is at the forefront of research on autophagy in podocytopathies world, which may be explained by the close collaboration among authors at these different institutions in China. Apart from the total number of citations, the average number of citations per item also reflects the quality of the publications and the academic impact of a country (25). Specifically, our analysis results show that France was the leading country according to ACI, followed by Australia and Germany. Whereas, China's average citation only ranked ninth, which may be related to the following factors in addition to major pioneer researchers and substantial financial support. Firstly, China's leading publication volume but relatively lower average citation rate in the field of autophagy in podocytopathies also suggests a large gap between the international influence of China and that in European and American countries. From a collaborative perspective as illustrated in Figure 2C, there is a lack of collaboration between China and those countries with high international influence in the field of autophagy in podocytopathies, which could be detrimental to the long-term development of academia. Another reason that cannot be ignored is that the prevalence of podocytopathies in China is much lower than that in Australia, American, Germany, France, and Japan (3). Due to the low incidence of podocytopathies in China, we may not have paid enough attention to the research of this kind of disease, leading to scientific research level lags far behind. Thus, strengthening the collaboration with these countries in this field of autophagy in podocytopathies would be meaningful. In terms of journal analysis, Front Pharmacol (IF = 5.99, Q1) and Cell Death Dis (IF = 9.71, Q1) published the most publications related to autophagy in podocytopathy research. In addition, several authoritative journals in the field of kidney disease, including the American Journal of Physiology-Renal Physiology and Kidney International, have also published several high-quality research findings in this field, suggesting the particular important role of autophagy in podocytopathies. Moreover, these above journals with high IFs and JCR categories can be the preferred submission choices for researchers who are dedicated to the study of autophagy in podocytopathies.

Given that references are the knowledge base for research, reference co-citation analysis can reflect the knowledge structure, trace the research evolution, as well as indicate current research hotspots of a certain research field (32, 45). Among those citations of autophagy in podocytopathies field, the most cited one is a research article published in the Journal of Clinical Investigation titled “Autophagy influences glomerular disease susceptibility and maintains podocyte homeostasis in aging mice,” which systematically investigated the exact role of podocyte autophagy deficiency in podocytopathies (21). This study confirmed for the first time that podocyte-specific deletion of autophagy-related 5 (Atg5) exhibited strongly increased susceptibility to models of glomerular disease, highlighting the importance of induced autophagy as a key homeostatic mechanism to maintain podocyte integrity (21). The second most cited one is the research article entitled “Impaired Podocyte Autophagy Exacerbates Proteinuria in Diabetic Nephropathy” published in Diabetes, which demonstrated that podocyte-specific autophagy-deficient mice aggravated podocyte loss and massive proteinuria in a high-fat diet (HFD)-induced diabetic model (41). These above two research articles are widely cited, primarily due to their pioneering research methods and both published in highly influential journals. Remarkably, it is worth noting that among the top 10 co-cited articles in the field of autophagy in podocytopathies, Japanese scholars contributed three, Chinese, American, and German scholars each contributed two, and French scholars contributed one, suggesting that strengthening cooperation with those above well-known scholars will greatly contribute to the development of autophagy in podocytopathies research. Furthermore, despite the existence of international cooperation, research in this field is mainly concentrated mainly focused on the United States, Europe countries, or other developed countries, highlighting the urgent need for a comprehensive international cooperation, so as to facilitate the better development of this field.

Keywords reflect the topic of an article, which can be used to analyze research progress, research hotspots, and novel directions in a certain scientific field (27). The co-occurrence of keywords analysis indicates that “diabetic nephropathy,” “autophagy,” “oxidative stress,” “injury,” and “endoplasmic-reticulum stress” have a relatively high frequency, suggesting that the association between autophagy and diabetic nephropathy has been one of the most prominent topics of autophagy in podocytopathies field. At present, the mechanisms regarding DKD-related impairment of podocyte autophagy are still under intensive investigation (16). For example, one previous study indicated that prolonged hyper-glycaemia *in vitro* or in the late stages of diabetes might suppress autophagy in podocytes, which contributed to the progression of DKD (40). Another study further showed that podocyte autophagy was significantly impaired in patients with type 2 diabetes, and that stimulation of cultured podocyte with serum from type 2 diabetes could inhibit autophagy (41). Since dysregulation of nutrient, energy-sensing pathways, as well as intracellular stress signals (mainly including reactive oxygen species, endoplasmic-reticulum stress and hypoxia) have all been involved in the regulation of podocyte autophagy in the pathogenesis of DKD (16, 42), pharmacological enhancement of autophagy will be beneficial to the treatment of podocytopathies. Therefore, developing small-molecule drugs targeting inhibition of the activation of the intracellular stress signals as well as restoring the reduced podocytes autophagy levels holds good potential for clinical application, which warrants further validation.

In addition, keywords with strong citation bursts are another critical indicator of hotspots and emerging trends in specific research field (25, 32). With time, it can be observed that the research focus has gradually shifted from “focal segmental glomerulosclerosis,” “podocyte function” to “therapeutic target,” and “NLRP3 inflammasome.” From the above analysis, we speculate that the future research hotspots of autophagy in podocytopathies field are related to identify novel therapeutic targets for the prevention and treatment of podocytopathies, as well as explore the role of inflammation in the progression of podocytopathies. A recent study reported that inhibition of NLRP3 inflammasome with MCC950, a NLRP3 inflammasome specific inhibitor, protected against podocyte damage through suppression of lipid accumulation in db/db mice (46). This suggests that MCC950 may be an effective drug for treating podocytopathies, which still needs more clinical experimental data verification. In the meantime, many other *in vivo* studies have focused on the therapeutic potential of autophagy activators in podocytopathies and promising therapeutic results in halting podocyte injury and ameliorating proteinuria (22, 47, 48). However, no clinical trial with autophagy regulators in podocytopathies has been conducted at the moment, mainly due to the lack of podocyte-directed drug delivery systems and difficulty in accurate therapeutic intervention on autophagy. Thus, developing podocyte-directed drug delivery systems and monitoring methods for changes in autophagy levels will promote the clinical application of autophagy regulators in podocytopathies. Despite the obvious difficulties, future research should include in-depth experimental research, validation of preclinical findings with samples from patients with podocytopathies, and the testing of the potential therapeutic targets that could modulate podocyte autophagy in clinical trials.

The current bibliometric analysis has the following four main shortcomings. Firstly, like the majority of bibliometric studies, we only obtained literature in the field of autophagy in podocytopathies from the WoSCC database, which may have ignored some relevant publications from other databases. Given that the WoSCC is considered to be the most commonly applied and recommended database for bibliometric analysis, data included in WoSCC can depict most of the information in a given field. Nevertheless, we still encourage future research should pay more attention to researches published in other literature databases, for example EBSCO, Google Scholar and MEDLINE, to identify additional publications, so as to comprehensively grasp the dynamic development trends of this research field. Secondly, due to the continuous updating of data in the WoSCC, the bibliometric analysis results typically fall behind the actual research situation. Therefore, the scientific trends of autophagy in podocytopathies research may change with time and bibliometric data updates. In the future research, we will continue to focus on autophagy in podocytopathies research field, hoping to help the relevant scholars better grasp the research structure and latest development trends. Thirdly, in comparison to conventional reviews, CiteSpace analysis may lack thoroughness (49, 50). Thus, it cannot clearly distinguish the first author from the corresponding author, nor can it distinguish between authors with the same name but different units, which may introduce biases in the results related to affiliated institutions and individual research outputs, resulting in some authors' academic contributions being underestimated or overestimated. Besides, there still remains defects with CiteSpace software in the unified parameter setting standards, which may yield to unexpected different analysis results during the software clustering process. Although there is still a lack of effective solutions to avoid these deficiencies, we firmly believe that the CiteSpace research team will continuously enhance this software and ultimately address these limitations while providing more precise analytical conclusions. Last but not the least, although the bibliometric analysis by employing specialized software is objective, the following interpretation of the results is somewhat subjective. Different researchers may have different cognitions and interpretations on even the same content. To this end, through multiple discussions among all authors, this study tries to overcome the subjective interpretation by individual. Despite of these shortcomings, in this study, all maps based on the retrieved data can intuitively present hotspots, evolution process, and development trends in the field of autophagy in podocytopathies, which can provide many important reference values for the newcomers to this research area.

## 5 Conclusion

Our study is the first time to use bibliometric analysis to illustrate the current status and emerging global trends on autophagy in podocytopathies research field, and has identified specific countries/regions, institutions, authors, as well as journals that have made significant contributions to this field during the past 15 years. Overall, the field of autophagy in podocytopathies research is still in the rapid ascending phase, and it can be seen that this area of research is likely to continue to expand for the next several decades. The leading countries are China, the United States, Germany, and Japan, with numerous collaborations and communications among authors from different countries/regions and institutions. The bibliometric analysis provides an objective and quantitative method for assessing the trends and leading edge of autophagy in podocytopathies, which provides a reference for scholars to further investigate the role of autophagy in podocytopathies as well as conduct clinical trial with autophagy regulators in podocytopathies.

## Data Availability

The raw data supporting the conclusions of this article will be made available by the authors, without undue reservation.
